# Gravity-Based Methods for Heading Computation in Pedestrian Dead Reckoning

**DOI:** 10.3390/s19051170

**Published:** 2019-03-07

**Authors:** Adi Manos, Itzik Klein, Tamir Hazan

**Affiliations:** Technion-Israel Institute of Technology, Haifa 32000, Israel; iklein@technion.ac.il (I.K.); Tamir.Hazan@technion.ac.il (T.H.)

**Keywords:** indoor navigation, smartphone sensors, gravity direction, heading estimation

## Abstract

One of the common ways for solving indoor navigation is known as Pedestrian Dead Reckoning (PDR), which employs inertial and magnetic sensors typically embedded in a smartphone carried by a user. Estimation of the pedestrian’s heading is a crucial step in PDR algorithms, since it is a dominant factor in the positioning accuracy. In this paper, rather than assuming the device to be fixed in a certain orientation on the pedestrian, we focus on estimating the vertical direction in the sensor frame of an unconstrained smartphone. To that end, we establish a framework for gravity direction estimation and highlight the important role it has for solving the heading in the horizontal plane. Furthermore, we provide detailed derivation of several approaches for calculating the heading angle, based on either the gyroscope or the magnetic sensor, all of which employ the estimated vertical direction. These various methods—both for gravity direction and for heading estimation—are demonstrated, analyzed and compared using data recorded from field experiments with commercial smartphones.

## 1. Introduction

Indoor navigation for pedestrians is essential for various location-based applications, such as commercial and emergency services. In the absence of Global Navigation Satellite System (GNSS) solution indoors, a common approach to handle this task is to use the inertial and magnetic sensors embedded in smartphones or other wearable devices, in a framework known as pedestrian dead reckoning (PDR) [1]. Typical PDR algorithms consist of estimating pedestrian’s step length and heading angle. These are combined, along with initial conditions, in order to calculate the pedestrian’s trajectory in a horizontal coordinate system. Detecting the step events and estimating their length is usually based on the accelerometer signals with bio-mechanical or empirical models. The pedestrian’s heading is estimated using the gyroscope and/or magnetic sensors. Other tasks related to PDR include the problem of vertical motion, such as identifying movements of pedestrian walking on stairs and using an elevator; or the challenge of incorporating map information into the dead reckoning scheme. Recently, it was shown that motion mode classification (pedestrian mode [2] and smartphone mode [3]) improves positioning accuracy in PDR by enabling a proper gain selection for the step length estimation phase.

In PDR algorithms, gravity direction estimation has a central role, although this is usually not mentioned explicitly in the literature. The direction of gravity can be regarded as the unit vector perpendicular to the local horizontal (typically North–East) plane, pointing vertically downwards. When expressed in the sensor frame of reference (that is, the device’s coordinate system), this vertical direction vector is generally time-varying due to rotational motion of the pedestrian. Once the gravity direction is estimated in this frame, it can be utilized to decompose any vector measurement into its vertical and horizontal components. This decomposition is essential for various PDR tasks, for example:

- Calculation of the horizontal turning rate, which is equivalent to the vertical projection of the three-axis gyroscope measurements, can be used for computing the heading angle.
- Extracting the horizontal acceleration signal out of the three-axis accelerometer measurements can be employed for identifying the dominant horizontal motion direction (that is usually associated with the forward direction).
- Several step detection and step length estimation methods require only the vertical acceleration signal.
- In order to use magnetic sensors for solving the heading angle, information on the horizontal plane as embedded in the gravity direction needs to be incorporated as well.

That is, accurate estimation of the gravity direction is critical for these tasks.

This paper is an extension of our initial work [4], in which several methods for gravity direction estimation and gyroscope-based heading determination were derived and compared. In this extended work, we present additional methods and new experimental results and analysis, as detailed in Section 1.2.

### 1.1. Related Work

A comprehensive survey on indoor navigation was given in [5] with an emphasis on employing inertial sensors for this purpose, while solutions that are specifically smartphone-based were surveyed in [6]. The problem of heading estimation is considered to be central in the PDR framework, since heading errors have a dominant impact on the overall positioning accuracy. A relatively recent work, [7], introduced a PDR solution using smartphones, which included heading estimation algorithm that logically selects how to blend gyroscope measurements with magnetic data, based on correlations between the two. Another approach that is common in the literature is to combine the gyroscope with additional assumptions on the motion characteristics. For example, the authors of [8], which addresses smartphone-based PDR, apply threshold-based turn detection algorithm on the gyroscope data, assuming that the heading angle changes only at distinctive turning events. Other works, such as [9], propose to employ the magnitude of gyroscope measurements for the calculation of turning angles. We refer the reader to additional related works on heading estimation in the Introduction section of [4].

Attitude of the sensor frame is closely related to the gravity direction, as the latter holds partial information on the former. Attitude estimation is investigated in a recent paper, [10], where various state-of-the-art algorithms for this task are detailed and compared. The context in their work is pedestrian-carried smartphones, even though they focus primarily on augmented reality applications.

### 1.2. Paper Contributions

In this work, we introduce and derive two approaches for gravity direction estimation, and utilize them as a baseline for heading determination. The first approach is based directly on the accelerometer measurements, while the second employs the gyroscope along with information on stationary intervals for relative attitude estimation. Additionally, by incorporating the estimated gravity direction, we derive and compare four different methods for computing the heading angle. The first one employs only gyroscopes and is based on the vertical component of the angular rate, as was proposed in [7] for example. The second one uses the magnitude of the angular rate vector, as was suggested in [9], yet herein we propose and implement a different computational approach to identify the direction of horizontal turning. Another gyroscope-based method presented here attempts to mitigate heading drifts, by discriminating straight path segments from curved ones and employing this information for dynamic bias compensation. Lastly, we also show detailed derivation of magnetic-based heading determination, which is computationally different from what we have seen in the literature (in [11] for example), since it directly incorporates the gravity unit vector, without explicit calculation of tilt angles; hence, in our magnetic-based method, trigonometric computations are replaced by algebraic ones.

The focus in this work is on the analytical derivation of these computational methods, as well as on qualitative analysis, comparisons and demonstrations using real-world experiments. It is noteworthy that the various approaches for heading estimation, as presented here, should be regarded as fundamental building blocks for PDR algorithms, rather than system-level solutions to the entire indoor navigation problem. In particular, we do not address absolute heading angle but merely relative ones, since the former requires additional information such as pedestrian forward direction in the sensor frame (as treated in [8], for instance). Moreover, we do not consider various cases of device pose in our experiments, but rather focus on a typical one (namely, smartphone in a pocket) for the purpose of demonstrating the proposed methods. Nevertheless, in the derivations, no assumption on a specific device pose is made, hence these methods can be equally applied to handle various device poses.

Within the scope of PDR research, the contributions of this paper are: (1) constituting a framework for gravity direction estimation while emphasizing the central role it has for various tasks in PDR; (2) providing detailed derivation of several fundamental models for heading computation, all of which rely on the estimated direction of gravity; (3) demonstrating and analyzing all of the various methods, based on field experiments with newly established methodology for obtaining the ground truth. In addition, at the end of this paper, we also present an empirical result regarding different forms of implementing sequential processing. This analysis is demonstrated on gyroscope data, but in fact is relevant to any tri-axis sensor, particularly those used in PDR.

The rest of this paper is organized as follows: Section 2 presents a detailed formulation of two methods for estimating the gravity direction. Section 3 shows the derivation of three approaches for gyroscope-based heading determination as well as one magnetic-based approach. Experimental results are analyzed and discussed in Section 4, and the conclusions are summarized in Section 5.

## 2. Methods for Gravity Direction Estimation

### 2.1. Problem Formulation

The gravity vector, denoted by $\vec{g}$, can be expressed in the local North–East–Down (NED) frame as $g^{n}={\left[\begin{array}{ccc} 0 & 0 & g_{0} \end{array}\right]}^{T}$, where $g_{0}$ is the magnitude of the local gravity and is measured in units of m/s^2^. Although some highly accurate gravity models may have different expressions for $g^{n}$ (such as non-zero north and east components), these differences can be safely neglected when dealing with PDR applications. In the sensor frame, the expression for the gravity vector depends on the orientation of the sensor relative to the NED frame and can be written as

(1) $$g^{s}=g_{0}\cdot \gamma^{s},$$

where we have defined $\gamma^{s}={\left[\begin{array}{ccc} \gamma_{x} & \gamma_{y} & \gamma_{z} \end{array}\right]}^{T}$ as the unit vector that represents the direction of gravity in the sensor frame. Denoting θ and ϕ as the pitch and roll angles of the sensor, respectively, they are related to $\gamma^{s}$ by

(2) $$\gamma^{s}={\left[\begin{array}{ccc} -\sin\theta & \sin\phi \cdot \cos\theta & \cos\phi \cdot \cos\theta \end{array}\right]}^{T},$$

based on the transformation matrix between NED frame and the sensor frame.

Thus, the problem of gravity direction determination can be formalized as the problem of computing or estimating the unit vector $\gamma^{s}$, which in general can be time-varying due to dynamic variations in the sensor’s orientation (e.g., for sensors carried by a pedestrian in motion).

### 2.2. Accelerometer-Based Method

The accelerometer is a three-axis sensor that measures the non-gravitational acceleration vector (also known as specific force), denoted by $\vec{f}$. Therefore, when the sensor is at rest, the true specific force equals the local gravity with an opposite sign. For an accelerometer that experiences motion with instantaneous acceleration $\vec{a}$, the specific force is

(3) $$\vec{f}=\vec{a}-\vec{g}.$$

This can be expressed in the sensor frame, using Label (1), as

(4) $$f^{s}\left(t\right)=a^{s}\left(t\right)-g_{0}\cdot \gamma^{s}\left(t\right),$$

where *t* is the instantaneous time and $a^{s}\left(t\right)$ is the vector of accelerations due to motion, expressed in the sensor frame. In the rest of the paper, we omit the s-superscript whenever it is clear that the vectors are expressed in the sensor frame.

Applying low-pass filtering to each axis of f(t) can reduce or eliminate the large temporal variations that are typically inherent in a(t) for a pedestrian in motion. Normally, these motion accelerations are expected to have approximately zero power density for the near-zero frequencies, otherwise the pedestrian would be constantly accelerating. An exception to this rule is when observing a(t) in a short time-window around some transient motion such as the beginning or stopping of walking, where acceleration or deceleration are expected.

Denoting $f_{p}\left(t\right)={\left[\begin{array}{ccc} f_{x,p}\left(t\right) & f_{y,p}\left(t\right) & f_{z,p}\left(t\right) \end{array}\right]}^{T}$ as the low-pass-filtered measurements and assuming the selected filter is linear, one can use Label (4) to write

(5) $$f_{p}\left(t\right)=a_{p}\left(t\right)-g_{0}\cdot \gamma_{p}\left(t\right),$$

where $a_{p}\left(t\right)$ and $\gamma_{p}\left(t\right)$ are the low-pass-filtered motion accelerations and gravity direction signals, respectively. Based on the above observation regarding pedestrian’s accelerations, we can assume that, if the selected cutoff frequency is low enough, then $a_{p}\left(t\right)\approx 0$, except for some short intervals of transient motion. Hence, during most of the time, the filtered accelerometer measurements satisfy

(6) $$f_{p}\left(t\right)\approx -g_{0}\cdot \gamma_{p}\left(t\right),$$

namely they approximate the filtered gravity signal. Finally, normalizing each vector measurement and inverting the sign should result in an estimation of the desired gravity direction in the sensor frame,

(7) $$\hat{\gamma }\left(t\right)=\frac{-f_{p}\left(t\right)}{∥f_{p}\left(t\right)∥}=\frac{-f_{p}\left(t\right)}{\sqrt{f_{x,p}^{2}\left(t\right)+f_{y,p}^{2}\left(t\right)+f_{z,p}^{2}\left(t\right)}}.$$

One of the trade-offs to be considered in the selection of the cutoff frequency of the filter is as follows: lower (higher) cutoff frequency will remove more (less) of the motion accelerations that “corrupt” the gravity component but will also result in slower (faster) response to changes in the true gravity direction, such as the periodic variations during the stride cycle or the change of sensor’s orientation when the pedestrian takes the device out of the pocket.

In this work, for the purpose of low-pass filtering, we decided to use an infinite impulse response (IIR) filter. This is in contrast to using simple moving average, which is basically a rectangular window finite impulse response (FIR) filter. The advantages of an IIR filter are well-known (e.g., [12]) in signal processing theory: it can achieve the same attenuation properties with much lower filter-order compared to the FIR filter, meaning its input-to-output delay is much shorter and it is also much simpler in terms of computations and memory usage. Specifically, we start with a 2nd-order transfer function of the form (based on the Butterworth design method for analog low-pass filters)

(8) $$H\left(s\right)=\frac{\omega_{c}^{2}}{s^{2}+\sqrt{2}\omega_{c}\cdot s+\omega_{c}^{2}},$$

where $\omega_{c}$ is the selected cutoff frequency in rad/s (and is related to $\tau_{c}=1/\omega_{c}$, known as the filter’s time constant). Then, to obtain the digital formulation of the IIR filter, one can use the bilinear transform $s=\frac{2}{T}\cdot \frac{1-z^{-1}}{1+z^{-1}}$, where *T* is the sampling interval of the sensor.

There exist several possible sources of error in the accelerometer-based method for gravity direction estimation. One obvious source is the inherent measurement errors of the sensor, which include random noise, bias and possibly also scaling and misalignment of the axes. Regarding the random noise, most of its power would be removed during the low-pass filtering since this noise naturally has very wide bandwidth. The other factors—particularly the bias term—might be dominant sources of error when dealing with low-cost sensors (which is the common case for PDR applications). Another source of error is the residual accelerations due to pedestrian motion that were not entirely removed by the low-pass filtering. These residuals might include temporally short components such as the forward accelerations at the beginning of walking or the lateral accelerations during turns. Lastly, the filtering process—aimed at removing the motion accelerations—inherently removes also part of the variations in the true gravity direction as observed in the sensor frame, particularly the periodic changes during the walking cycle, in which the pedestrian-carried device exhibits rotational motion. In other words, these true variations might be “averaged-out” by the filter, depending on how low the cutoff frequency was selected. Dealing with this source of error is the motivation behind the other method for gravity direction estimation, which is presented next.

### 2.3. Gyroscope-Accelerometer Fusion Method

A gyroscope measures the angular rate around its three sensitive axes. The sensor may exhibit angular rate resulting both from the pedestrian’s motion and from the rotational motion of the Earth. The latter can be safely neglected for our purpose, as it is much smaller than the accuracy of typical gyroscopes used in PDR applications.

In the context of estimating gravity direction, the gyroscope measurements provide information that is useful in tracking the rotational dynamics of the sensor frame during the pedestrian’s walking cycle. This information may compensate for some of the limitations of the accelerometer-based method as was described in Section 2.2. The method that will be presented here utilizes this information within a Kalman Filter framework, by combining it with the accelerometer data. This filter is similar in some ways to the one proposed in [8] for device attitude estimation, but here we incorporate a different measurement model as well as different initialization scheme (in particular, we do not assume the sensor frame to have any specified initial orientation).

First, in order to initialize the filter, the method assumes the existence of a short stationary time period of the sensors prior to the pedestrian’s walking phase. Such an interval can naturally occur, for example when the pedestrian is standing still or when the device is initially placed on a table. The problem of automatic detection of stationary conditions is beyond the scope of this paper, but is relatively simple to solve, for instance based on thresholding the empirical variance of the accelerometer and gyroscope measurements in a sliding window (e.g., similarly to [13]). The inertial measurements during the stationary interval can be used for two purposes:

1. The average angular rate in each of the gyro’s axes can be treated as the residual bias error because one can expect these measurements to be zero when the sensor is at rest. This estimated bias can be removed from any future gyro’s measurements, thus reducing possible drifts when integrating them.
2. Determining the gravity direction based on the accelerometer alone is more reliable when the sensor is stationary, since no motion accelerations are present (though bias and other sensor errors still degrade the accuracy). In this case, we suggest averaging the measurements within the stationary interval instead of applying the low-pass filter, thus obtaining a single gravity direction estimation, ${\hat{\gamma }}_{s}$, that will be used as initialization for the procedure described next.

Starting from the end of the stationary interval at time $t=t_{s}$, we will integrate the gyro’s raw measurements, $\omega =\left[\begin{array}{ccc} \omega_{x} & \omega_{y} & \omega_{z} \end{array}\right]$, to dynamically compute the attitude of the sensor’s instantaneous frame relative to its initial frame from time $t_{s}$. This attitude state will be represented by the quaternion

(9) $$q\left(t\right)={\left[\begin{array}{cc} q_{v}^{T} & q_{c} \end{array}\right]}^{T},$$

with vector part $q_{v}\in R^{3}$ and scalar part $q_{c}\in R$. It is related to the gyro rate by the kinematic equation

(10) $$\frac{d}{dt}q\left(t\right)=\frac{1}{2}\Omega \left(\omega (t)\right)\cdot q\left(t\right),$$

where Ω is the 4 × 4 matrix defined by

(11) $$\Omega \left(\omega \right)=\left[\begin{array}{cc} -\left[\omega \times \right] & \omega \\ -\omega^{T} & 0 \end{array}\right]$$

with $\left[\omega \times \right]$ being the skew-symmetric matrix of the vector ω. Given the quaternion state at time instance *t*, we can express the previously-computed unit-vector $\gamma_{s}$ in the current sensor frame by applying the transformation matrix C(q(t)), which is related to the quaternion by

(12) $$C\left(q\left(t\right)\right)=\left(q_{c}^{2}-q_{v}^{T}q_{v}\right)I_{3}-2q_{c}\left[q_{v}\times \right]+2q_{v}q_{v}^{T}$$

($I_{n}$ is the n×n identity matrix). Thus, the estimated gravity direction at time *t* is computed as

(13) $$\hat{\gamma }\left(t\right)=C\left(q\left(t\right)\right)\cdot \hat{\gamma_{s}}.$$

Since the gyroscope measurements inherently contain errors, such as random noise and bias, the simple integration described above will cause the computed attitude state to drift with time. For this reason, we propose to employ the gravity information obtained from the accelerometer in (7), by using a fusion scheme based on the Multiplicative Extended Kalman Filter (MEKF). This type of filter is presented in [14] for an attitude estimation problem, where:

- The attitude state of the sensor frame, relative to some reference frame, is represented by a quaternion, *q*. The estimation error quaternion, denoted δq, describes the residual rotation from the estimated sensor frame to the true one, which can be formalized as
  (14) $$C\left(q\right)=C\left(\delta q\right)\cdot C\left(\hat{q}\right),$$
  where $\hat{q}$ is the estimated attitude quaternion. For small attitude errors, δq can be linearized as
  (15) $$\delta q\approx \left[\begin{array}{cc} \delta \theta^{T}/2 & 1 \end{array}\right],$$
  with $\delta \theta \in R^{3}$ representing the error state (it describes the angular error and its rotation axis).
- The process model of the filter is driven by the angular rate measurements of the gyro, as in (10). The linearized formulation of the attitude error dynamics is
  (16) $$\frac{d}{dt}\delta \theta =-\left[\omega \times \right]\cdot \delta \theta +\delta \omega ,$$
  where δω is the gyro’s measurements error vector.
- The measurement model of the filter uses direction vectors that are measured continuously in the sensor frame, with prior information about their static representation in the reference frame. For a single direction vector, *y*, with additive measurement error vector, *v*, the model is
  (17) $$y^{s}=C\left(q\right)\cdot y^{r}+v,$$
  where $y^{s}$ and $y^{r}$ are the representations of *y* in the sensor frame and in the reference frame, respectively. The error vector is assumed to have covariance matrix $R=\sigma^{2}I_{3}$.

**Figure 1:**
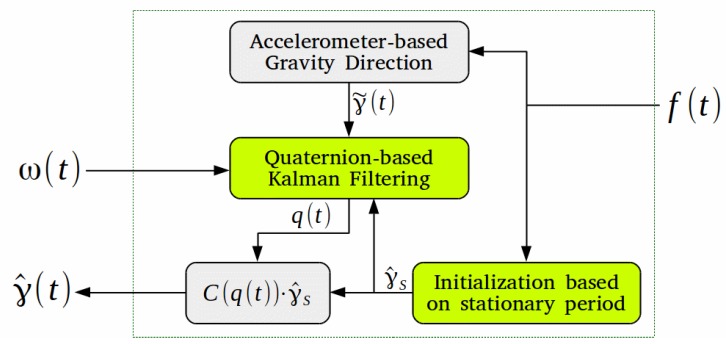
Structure of the filter for gravity direction estimation. Input signals from the accelerometer and gyroscope are f(t) and ω(t), respectively, while the output $\hat{\gamma }\left(t\right)$ is the estimated gravity unit vector.

In this paper, the direction vector measurements are those obtained by the accelerometer-based method introduced in Section 2.2, while the reference frame is the stationary sensor frame from time $t=t_{s}$, in which the direction vector is represented by ${\hat{\gamma }}_{s}$. The structure of the proposed filter is shown in Figure 1. For the discrete time formulation of the filter, we will use the subscript *k* to denote any variable at the kth time step, with k=0 referring to the initial time, $t_{s}$. The main stages for dynamic estimation of the gravity direction using the MEKF are as follows:

1. **Initialization:** Given an initial stationary time interval, $t\in [0,\;t_{s}]$, we first compute the average specific force vector, $f_{\mathrm{avg}}$, by averaging the accelerometer measurements (axis-wise) in that interval. Then, using (7) with $f_{\mathrm{avg}}$ replacing $f_{p}\left(t\right)$, the reference gravity direction in the stationary sensor frame, ${\hat{\gamma }}_{s}$, can be computed. In addition, the attitude quaternion state and the error covariance matrix need to be initialized. At $t=t_{s}$, the instantaneous sensor frame is identical to the stationary one, thus the initial state estimate is the identity quaternion,
  (18) $${\hat{q}}_{0}^{+}={\left[\begin{array}{cccc} 0 & 0 & 0 & 1 \end{array}\right]}^{T}.$$
  Assuming an initial angular error with variance $\sigma_{\mathrm{init}}^{2}$, the covariance *P* of δθ can be initialized as
  (19) $$P_{0}^{+}=\sigma_{\mathrm{init}}^{2}\cdot I_{3}.$$
2. **Propagation step:** The discretization of (10) with time step Δt, under the approximation of constant ω(t) during each time step, is as follows:
  (20) $$\begin{array}{cc} {\hat{q}}_{k+1}^{-} & =F_{k}\cdot {\hat{q}}_{k}^{+}, \end{array}$$
  (21) $$\begin{array}{cc} F_{k} & \equiv I_{4}\cos\left(\beta_{k}\right)+\Omega \left(\omega_{k}\right)\cdot \frac{\sin(\beta_{k})}{∥\omega_{k}∥}, \end{array}$$
  (22) $$\begin{array}{cc} \beta_{k} & \equiv ∥\omega_{k}∥\cdot \Delta t/2. \end{array}$$
  Even though Equation (20) preserves the unit norm of the quaternion, its numerical implementation should include normalization of the calculated ${\hat{q}}_{k+1}^{-}$, in order to avoid accumulating quantization errors. Next, the propagation of the error covariance is computed as
  (23) $$P_{k+1}^{-}=\Phi_{k}P_{k}^{+}\Phi_{k}^{T}+Q_{d},$$
  where $\Phi_{k}$ and $Q_{d}$ are the transition matrix and noise covariance of the discrete time error dynamics, respectively. Based on (16), the 1st-order approximation of $\Phi_{k}$ is
  (24) $$\Phi_{k}=I_{3}-\left[\omega_{k}\times \right]\cdot \Delta t,$$
  and assuming the gyro’s white noise has power spectral density (PSD) of $\sigma_{g}^{2}$$\left[{\mathrm{rad}}^{2}\times \mathrm{Hz}\right]$, the approximation of $Q_{d}$ is
  (25) $$Q_{d}=\sigma_{g}^{2}\cdot \Delta t\cdot I_{3}.$$
3. **Measurement update step:** When a measured gravity direction, $\tilde{\gamma }$, is given at some time instance along with its covariance matrix, *R*, we first compute the measurement prediction using the prior estimate of the attitude quaternion, ${\hat{q}}^{-}$:
  (26) $${\hat{\gamma }}^{-}=C\left({\hat{q}}^{-}\right)\cdot {\hat{\gamma }}_{s},$$
  from which the linearized measurement matrix can be calculated as
  (27) $$H=\left[\left({\hat{\gamma }}^{-}\right)\times \right].$$
  Then, based on the prior covariance, $P^{-}$, the Kalman gain matrix is
  (28) $$K=P^{-}H^{T}{\left(HP^{-}H^{T}+R\right)}^{-1}$$
  and the estimated attitude error vector is
  (29) $$\delta \hat{\theta }=K\cdot \left(\tilde{\gamma }-{\hat{\gamma }}^{-}\right).$$
  The quaternion attitude state is updated by
  (30) $${\hat{q}}^{+}={\hat{q}}^{-}+S\left({\hat{q}}^{-}\right)\cdot \delta \hat{\theta }/2,$$
  where the 4x4 matrix *S* is defined as
  (31) $$S\left(q\right)\equiv \left[\begin{array}{c} q_{c}I_{3}+\left[q_{v}\times \right]\\ -q_{v}^{T} \end{array}\right]$$
  and the resulting quaternion needs to be normalized. Finally, the covariance matrix is updated as usual by
  (32) $$P^{+}=\left(I_{3}-KH\right)P^{-}{\left(I_{3}-KH\right)}^{T}+KRK^{T}.$$

In contrast to the method presented in Section 2.2, for which there is only a single design parameter (namely the cutoff frequency of the low-pass filter), the current method requires the tuning of additional parameters for the MEKF, as follows: (1) the initial variance, $\sigma_{\mathrm{init}}^{2}$, which should measure the uncertainty in the estimation of ${\hat{\gamma }}_{s}$ as a result of systematic errors (mainly bias) of the accelerometer; (2) the process noise PSD, $\sigma_{g}^{2}$, which should be based on the gyroscope’s specifications but in practice requires some tuning to account for model uncertainty; and (3) the measurements covariance matrix, *R*. In order to simplify the tuning of the latter parameter, one can assume it has the form $R=\sigma_{a}^{2}I_{3}$ which requires tuning only the scalar variance $\sigma_{a}^{2}$ (in general, though, $\sigma_{a}^{2}$ can be time-varying, for instance, as a function of the empirical variance of the gravity direction measurements in a sliding window).

## 3. Methods for Heading Computation

The estimated gravity direction can be utilized for the problem of computing the heading angle based on either the gyroscope or the magnetometer measurements. In this section, we describe four different models for this purpose—three of which are based on the gyroscope, while the last one employs the magnetic sensor.

### 3.1. Preliminary Remark on Heading Definition

Before we describe each of these models, it is important to make a clear distinction between two possible types of heading angle in the context of pedestrian navigation. First, consider the heading of the sensor frame (namely the device carried by the pedestrian), which is a direct outcome of the angular motions experienced by the device and measured by these sensors. This heading angle does not take into account the relative orientation of the sensor frame with respect to the pedestrian’s body, while in fact this information is necessary for calculating the trajectory, as part of the dead reckoning process. Thus, the second type of heading angle is the one associated with the trajectory rather than the device. Moreover, even if the sensor frame is fixed on the pedestrian, it is still possible that the trajectory heading would change during walking, as a result of dynamic variations in the direction of motion relative to the forward axis of the pedestrian’s body (for example, when the pedestrian is walking partially sideways).

Within the scope of this work, we assume for simplicity that the trajectory heading differs from the device heading by a constant offset. Following this assumption, the methods for heading computation presented in this section attempt to achieve estimation of the relative heading of the device.

### 3.2. Vertical Angular Rate Component

The proposed gyroscope-based methods for heading computation employ the vertical component of the angular rates vector, which can be extracted using the estimated direction of gravity. Denoting this vertical projection of the gyroscope by $\omega_{v}$, its calculation at any instance of time can be done by

(33) $$\omega_{v}\left(t\right)=\hat{\gamma }{\left(t\right)}^{T}\omega \left(t\right),$$

where ω(t), the 3D vector of angular rates, should be obtained after appropriate filtering of the gyroscope’s raw measurements, in order to reduce the effects of random noise and high-frequency disturbances. Based on the right-hand rule, $\omega_{v}$ measures the pedestrian’s turning rate in the horizontal plane, where positive (negative) rate values indicate turning to the right (left). Thus, one approach for computing the change in heading is to directly integrate $\omega_{v}\left(t\right)$,

(34) $$\psi \left(t\right)-\psi_{s}=\int_{t_{s}}^{t_{f}}\omega_{v}\left(\tau \right)d\tau ,$$

where $\left[t_{s},t_{f}\right]$ is the time interval of interest (e.g., start and end of the scenario) and $\psi \left(t\right)-\psi_{s}$ is the heading angle in radians at time *t* relative to its initial value (namely, the accumulated change in heading).

### 3.3. Signed-Magnitude Angular Rate

Another approach for this task is to use the *magnitude* of the angular rate vector,

(35) $$\omega_{m}\left(t\right)\equiv \sqrt{\omega_{x}^{2}\left(t\right)+\omega_{y}^{2}\left(t\right)+\omega_{z}^{2}\left(t\right)},$$

as was suggested in [9], for example. The motivation behind this is as follows:

- The magnitude of vectors, when ignoring measurement errors, is independent of the frame in which they are measured, hence $\omega_{m}$ does not depend on the sensor’s orientation. In practice, however, systematic errors such as bias will cause the measured $\omega_{m}$ to have some dependence on the orientation.
- Although the turning rate of interest is disturbed by other angular rates, due to pedestrian’s motion, appropriate pre-filtering of ω(t) is expected to eliminate most of them, so that the magnitude of the filtered measurements should approximate the true horizontal turning rate.

Discriminating between left and right turns requires the sign of $\omega_{m}\left(t\right)$ to be determined for every instance of time. In [9], this was achieved by finding which axis (*x*, *y* or *z*) of the accelerometer is most dominant, and using the sign of the gyroscope in that axis to determine the direction of turning. Such a method might not be robust to situations in which two different axes are similarly dominant (for instance, when a smartphone is tilted $45^{\circ }$, the gravity appears equally on both the *y*-axis and *z*-axis). We suggest a different approach to distinguish left turns from right turns, by directly observing the sign of $\omega_{v}\left(t\right)$ and calculating the *signed-magnitude* rate signal

(36) $$\omega_{sm}\left(t\right)\equiv \mathrm{sgn}\left(\omega_{v}\left(t\right)\right)\cdot \omega_{m}\left(t\right),$$

where sgn(·) is defined as ±1 for positive/negative inputs and 0 otherwise. Finally, computation of the heading with this approach is performed as in Equation (34), but with $\omega_{v}$ replaced by $\omega_{sm}$.

### 3.4. Dynamic Turn Rate Bias Compensation

The main problem in gyroscope-based heading is the potential accumulation of errors in the integral calculation of Equation (34), i.e., angular drifts due to systematic errors. In this work, we propose a way of mitigating it, by assuming that some of the time the pedestrian walks in approximately straight path: during such segments, the average horizontal turning rate is expected to be ideally zero, hence any offset can be regarded as a bias in the computed $\omega_{v}$. In order to detect straight path segments, we apply a thresholding mechanism on the bias-compensated rate signal. The proposed method is implemented as a filter, which maintains an estimated bias b∈R, and a logical state s∈{0,1} indicating a straight segment. This filter is summarized as follows:

1. Initialize $b_{0}=0$ and $s_{0}=1$.
2. At time step $t_{k}$, given the measured $\omega_{v,k}$,
  (a) Update the bias estimation by
    (37) $$b_{k}=\left\{\begin{array}{cc} b_{k-1} & ,s_{k-1}=0,\\ \lambda \cdot b_{k-1}+\left(1-\lambda \right)\cdot \omega_{v,k} & ,s_{k-1}=1. \end{array}\right.$$
    Here, $\lambda =\exp(-\Delta t/T_{b})$, with $T_{b}=30$ [s] being a time-constant of this 1st-order filtering of the $\omega_{v}$ measurements; Δt is the sampling interval.
  (b) Update the state indicator by thresholding with $R_{\max}=10$ [deg/s],
    (38) $$s_{k}=\left\{\begin{array}{cc} 1, & =\left|\omega_{v,k}-b_{k}\right|\leq R_{\max},\\ 0, & \mathrm{otherwise}. \end{array}\right.$$
3. The corrected turning rate at time $t_{k}$ is calculated simply as
  (39) $$\omega_{v,k}^{c}=\left\{\begin{array}{cc} \omega_{v,k}-b_{k} & ,s_{k}=0,\\ 0 & ,s_{k}=1. \end{array}\right.$$

The relatively long time-constant above, $T_{b}$, is meant for two purposes: (1) producing a long accumulation of $\omega_{v}$ samples for averaging; (2) avoiding false identification of real turning rates as measurement bias.

### 3.5. Magnetometer-Based Heading Computation

The magnetometer is a three-axis sensor that measures the ambient magnetic field, which is a composition of the Earth’s geomagnetic field and local magnetic disturbances (due to ferro-magnetic materials, for example). In this section, we treat the computational problem of combining geomagnetic field vector with gravity direction vector, for the purpose of device heading angle calculation. Though the treatment of local disturbances is important in many real-world scenarios, we do not consider it here.

We use $m^{n}$ to denote the vector of geomagnetic field in the local NED frame. Its magnitude is $m_{0}$ and its direction deviates from the geographic north by declination angle α and inclination angle β,

(40) $$m^{n}=m_{0}\cdot {\left[\begin{array}{ccc} \cos\beta \cos\alpha & \cos\beta \sin\alpha & \sin\beta \end{array}\right]}^{T}.$$

Assuming the sensor frame is rotated relative to the NED frame by the three Euler angles (ϕ,θ,ψ), one can express the geomagnetic field vector in the sensor frame as

(41) $$m^{s}=\left[\Phi \right]\cdot \left[\Theta \right]\cdot \left[\Psi \right]\cdot m^{n},$$

where [Ψ],[Θ],[Φ] are the following orthonormal rotation matrices:

(42) $$\left[\Psi \right]=\left[\begin{array}{ccc} \cos\psi & \sin\psi & 0\\ -\sin\psi & \cos\psi & 0\\ 0 & 0 & 1 \end{array}\right],\;\left[\Theta \right]=\left[\begin{array}{ccc} \cos\theta & 0 & -\sin\theta \\ 0 & 1 & 0\\ \sin\theta & 0 & \cos\theta \end{array}\right],\;\left[\Phi \right]=\left[\begin{array}{ccc} 1 & 0 & 0\\ 0 & \cos\phi & \sin\phi \\ 0 & -\sin\phi & \cos\phi \end{array}\right].$$

By denoting the unit vectors of the geomagnetic field as $\mu^{n}=m^{n}/m_{0}$ and $\mu^{s}=m^{s}/m_{0}$, and by using the orthonormality property, we derive from (41):

(43) $${\left[\Theta \right]}^{T}\cdot {\left[\Phi \right]}^{T}\cdot \mu^{s}=\left[\Psi \right]\cdot \mu^{n}.$$

In the last equation, the left-hand side is related to the gravity direction and the magnetic north direction in the sensor frame; while the right-hand side represents the unknown heading angle, ψ, as well as the declination and inclination angles of the local geomagnetic field. These latter angles, α and β, are mapped empirically as part of the World Magnetic Model (WMM) [15] in which they are given as a function of latitude and longitude.

From what we have observed in the literature, e.g., [11], the heading angle is calculated based on explicit estimation of the tilt angles θ and ϕ. In what follows, we derive a different solution that directly employs the estimated gravity unit vector, without the need to apply trigonometric functions with respect to these tilt angles.

Looking back at Equation (2), we obtain the following relations between the components of the gravity direction vector $\left(\gamma_{x},\gamma_{y},\gamma_{z}\right)$ and the tilt angles θ and ϕ:

(44) $$\begin{array}{c} \sin\theta =-\gamma_{x}, \end{array}$$

(45) $$\begin{array}{cc} \gamma_{y}^{2}+\gamma_{z}^{2}=\cos^{2}\theta \;\Rightarrow \; & \cos\theta =\sqrt{\gamma_{y}^{2}+\gamma_{z}^{2}}\equiv \gamma_{yz}, \end{array}$$

(46) $$\begin{array}{c} \sin\phi =\frac{\gamma_{y}}{\cos\theta }=\frac{\gamma_{y}}{\gamma_{yz}}, \end{array}$$

(47) $$\begin{array}{c} \cos\phi =\frac{\gamma_{z}}{\cos\theta }=\frac{\gamma_{z}}{\gamma_{yz}}. \end{array}$$

Writing the right-hand side of (43) explicitly, using well-known trigonometric identities, we have

(48) $$\left[\Psi \right]\cdot \mu^{n}=\left[\begin{array}{ccc} \cos\psi & \sin\psi & 0\\ -\sin\psi & \cos\psi & 0\\ 0 & 0 & 1 \end{array}\right]\cdot \left[\begin{array}{c} \cos\beta \cos\alpha \\ \cos\beta \sin\alpha \\ \sin\beta \end{array}\right]=\left[\begin{array}{c} \cos\beta \cdot \cos(\psi -\alpha )\\ -\cos\beta \cdot \sin(\psi -\alpha )\\ \sin\beta \end{array}\right].$$

The tilt rotation matrix ${\left[\Theta \right]}^{T}\cdot {\left[\Phi \right]}^{T}$ from the left-hand side of (43) can be written explicitly as follows, where we omit the third row of the matrix for a reason that will be clear soon:

(49) $$A\equiv {\left[\Theta \right]}^{T}\cdot {\left[\Phi \right]}^{T}=\left[\begin{array}{ccc} \cos\theta & \sin\theta \sin\phi & \sin\theta \cos\phi \\ 0 & \cos\phi & -\sin\phi \\ \cdot & \cdot & \cdot \end{array}\right].$$

Then, using (44)–(47), it can be re-written as

(50) $$A=\frac{1}{\gamma_{yz}}\left[\begin{array}{ccc} \gamma_{yz}^{2} & -\gamma_{x}\cdot \gamma_{y} & -\gamma_{x}\cdot \gamma_{z}\\ 0 & \gamma_{z} & -\gamma_{y}\\ \cdot & \cdot & \cdot \end{array}\right].$$

Substituting (48) and (50) into (43), by denoting $\mu^{s}=\left[\begin{array}{ccc} \mu_{x} & \mu_{y} & \mu_{z} \end{array}\right]$, we obtain

(51) $$\begin{array}{cc} \gamma_{yz}^{2}\mu_{x}-\gamma_{x}\left(\gamma_{y}\mu_{y}+\gamma_{z}\mu_{z}\right) & =\gamma_{yz}\cos\beta \cdot \cos\left(\psi -\alpha \right), \end{array}$$

(52) $$\begin{array}{cc} \gamma_{y}\mu_{z}-\gamma_{z}\mu_{y} & =\gamma_{yz}\cos\beta \cdot \sin\left(\psi -\alpha \right). \end{array}$$

Therefore, applying the four-quadrant arctangent function on these last two equations, the common term $\left(\gamma_{yz}\cos\beta \right)$ vanishes and we have:

(53) $$\arctan2\left(\gamma_{y}\mu_{z}-\gamma_{z}\mu_{y}\;,\;\gamma_{yz}^{2}\mu_{x}-\gamma_{x}\left(\gamma_{y}\mu_{y}+\gamma_{z}\mu_{z}\right)\right)=\psi -\alpha ,$$

which is the heading angle relative to magnetic north. Obtaining the absolute heading relative to geographic north can be done by compensating the geomagnetic declination angle α based on the WMM. (For a typical scenario of pedestrian navigation—in which the traveled distance does not reach more than few kilometers—one can consider α as approximately constant.)

To summarize this subsection, once the gravity direction vector $\gamma^{s}$ and the geomagnetic field $m^{s}$ are estimated (or measured) in the sensor frame, the heading angle of the device can be computed using Equation (53), with $\mu^{s}$ obtained by normalizing the $m^{s}$ vector.

## 4. Experimental Results and Analysis

The various methods that have been developed in this paper—both for gravity direction estimation and for heading computation—are summarized in Table 1, including the related sub-section for each method. In order to analyze and compare them, we performed several experiments in natural settings, using commercial smartphones. The results shown in this section demonstrate these methods when applied to an experiment with a Samsung Galaxy A5 device (Seoul, Korea); previous results using other devices and experiments were presented in [4]. The inertial and magnetic sensors on the Galaxy A5 were recorded at a 100[Hz] sampling rate and later analyzed offline on a PC.

### 4.1. Ground Truth Heading

Obtaining ground truth for the heading angle in indoor environments is not trivial and usually requires dedicated infrastructure during experimental setup. In our previous work [4], we assumed the indoor corridors are perpendicular to one another, and extracted the true heading profile according to this assumption. Here, we have developed a new methodology for obtaining a reasonable ground truth heading, composed of the following steps:

1. Conducting the experiments in natural outdoor environment with open sky-view, which allows GNSS measurements with satisfactory accuracy.
2. Designing the walking path as a sequence of straight segments connected by short curves; this imitates a typical indoor environment made of straight corridors.
3. In addition, based on such walking paths, we can obtain relatively accurate ground truth, by averaging the GNSS-based heading data during each straight segment.

It is important to emphasize that these outdoor experiments share the same pedestrian dynamics as typical indoor walking experiments, and that the GNSS data is only used here as a reference for evaluating the different methods.

An error analysis of GNSS heading measurements is provided in [16]. It shows that the standard deviation (STD) of the GNSS heading, $\psi^{GNSS}$, has the following relation with the GNSS velocity, $V^{GNSS}$:

(54) $$\mathrm{STD}\left(\psi^{GNSS}\right)=\frac{\mathrm{STD}(V^{GNSS})}{V^{GNSS}}.$$

Assuming a typical STD of a single GNSS velocity measurement error to be around 0.1[m/s], with a typical walking speed of 1.5[m/s], results in an approximated value of 3.8[deg] for the STD of a single GNSS heading measurement error. When averaging over a sequence of *N* measurements (and assuming for simplicity that they are statistically independent), the error STD drops by a factor of $\sqrt{N}$. Therefore, for a straight walking path of 15–20 s, for instance, with a GNSS sampling rate of 1 Hz, we obtain a rough estimation of 0.85−−1.0[deg] for the error STD in the averaged GNSS heading angle.

### 4.2. Gravity Direction Results

For gravity direction estimation, low-pass filtering of the accelerometer was done using a 2nd-order IIR filter as described in Section 2.2, with a time-constant τ=0.4 s (which is equivalent to a cutoff frequency of approximately 0.398Hz). The parameters for gyro-accelerometer fusion scheme, as was described in Section 2.3, were set as follows: standard deviation $\sigma_{\mathrm{init}}=1.0\;\left[\deg\right]$ for the initial angular error; process noise with PSD of $\sigma_{g}^{2}=1.0\;\left[\deg^{2}\times \mathrm{Hz}\right]$; and a constant standard deviation $\sigma_{a}=9.0\;\left[\deg\right]$ for constructing the measurement covariance matrix, $R=\sigma_{a}^{2}I_{3}$. In addition, the raw gyroscope measurements during an initial static time interval were used to estimate a constant bias vector (simply by averaging), which in turn was reduced from these measurements along the entire duration of the experiment; in the case being analyzed, the calculated bias values were (−1.235,−0.555,1.774) [deg/s]. Regarding the magnetic data, it should be noted that we used its calibrated version as supplied by the Galaxy A5 device. Lastly, both gyroscope and magnetometer data were preprocessed with a 2nd-order low-pass filter using time-constant of 0.4 [s] (which is equivalent to a 0.398 [Hz] cutoff frequency).

**Figure 2:**
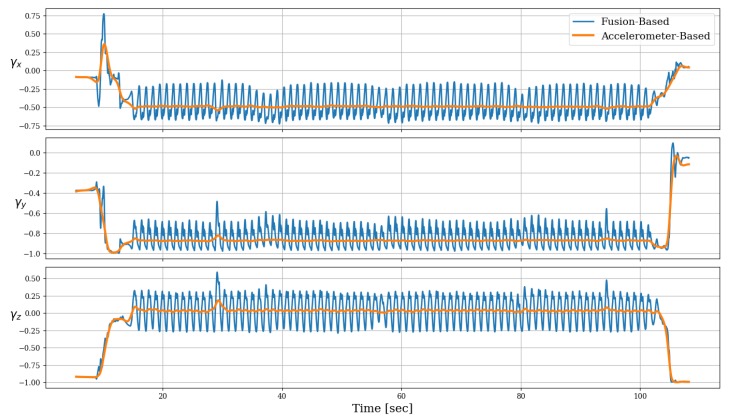
Estimated gravity direction along time, comparing the accelerometer-based method with the gyroscope-accelerometer fusion method.

Figure 2 shows the three components of the gravity direction vector in the sensor frame, as computed by each of the two methods, namely—the accelerometer-based and the fusion-based methods. In this experiment, the device was initially held stationary in hand, with the screen facing approximately upwards; this is indicated in the graphs, which show that the direction of gravity is mainly present in the negative *z*-axis at the beginning. Then, the device was placed in the trousers’ pocket, after which the pedestrian started a normal walking. At the end of the experiment, it was taken out of the pocket and held in hand again.

During walking, the gravity direction as estimated by both methods roughly aligns with the negative *y*-axis; but compared to the steady direction vector when using only accelerometer, fusion with the gyroscope shows large fluctuations in that signal. These reflect the angular motion of the leg during walking cycle, which causes the gravity unit-vector to change periodically in the sensor frame. The absence of these periodic changes in the accelerometer-based method confirms our notion from Section 2.2 that they are “averaged-out” as a result of the necessary low-pass filtering of the accelerometer signal. In addition, any transient event such as the one observed immediately after the stationary state, appears in fusion-based method with more subtle details.

There is no obvious way to extract the ground truth of the gravity direction vector, at least in any real world setting involving unconstrained smartphones, primarily since this unit vector changes dynamically within the walking cycle. Therefore, in order to compare the accuracy of different methods for gravity direction estimation, we shall analyze them in terms of their influence on the relative heading angle, for which an approximate ground truth is more easily obtained.

### 4.3. Heading Angle Results

**Figure 3:**
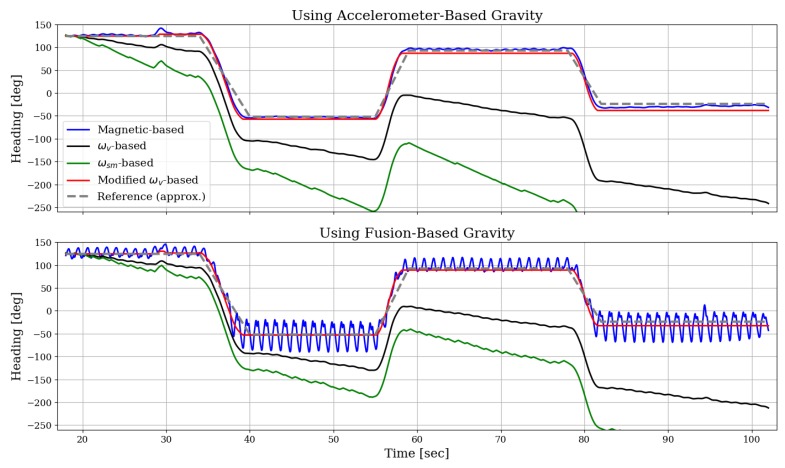
Comparing estimated heading profiles as obtained by the magnetic-based and the three gyroscope-based methods. Upper and lower panes show the results when each of the gravity direction estimation methods is applied.

Figure 3 shows the heading for the entire walking duration of the same experiment. The dashed gray line is the approximated reference based on the GNSS heading data. All other lines in this figure show estimated results obtained by the various methods. Recall that we only attempt to estimate the relative change in heading with respect to its initial value; therefore, we applied an angular offset to each estimated heading profile such that it would initially match the reference. The upper and lower panes of this figure are used here to demonstrate how the gravity direction estimation impact the resulting heading profiles. It can be seen that numerical integration of the vertical or signed-magnitude angular rate—denoted in the legend as $\omega_{v}$-based or $\omega_{sm}$-based, respectively—both result in a quickly-drifting heading error. In contrast, when applying dynamic bias-compensation on the vertical angular rate (as proposed in Section 3.4 and indicated here as “Modified $\omega_{v}$-based”), most of the drift is eliminated: the error at the end of this experiment—after approximately 85 s—is less than 15 degrees when using the accelerometer-based gravity, and less than nine degrees for the fusion-based gravity (note that these are also the maximal error values reached during the entire experiment). This gyroscope-based method is therefore comparable to the magnetic-based heading, which shows an error of 5–6 degrees, on average, during the last 20-s segment. Additionally, when the fusion-based gravity direction method is employed—rather than the accelerometer-based one—it seems that the two drifting heading profiles have somewhat reduced drift rate (though it is not necessarily a general consequence). On the other hand, for magnetic-based heading, it seems that using the accelerometer-based gravity direction produces better, less fluctuating signal. A summary of these results is provided in Table 2, comparing all various combinations of gravity direction and heading computation methods, in terms of the heading error.

**Figure 4:**
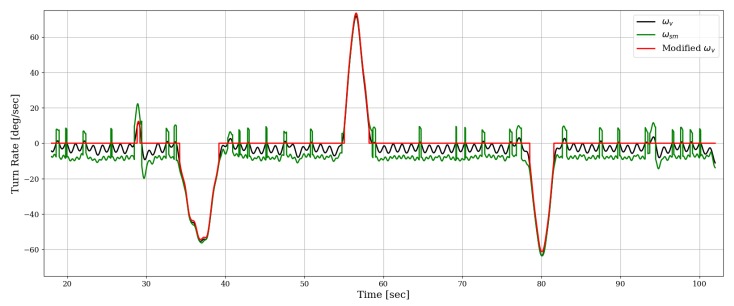
Comparing turn rate profiles as obtained by three gyroscope-based methods. The gravity direction here is estimated based on the accelerometer only.

For deeper understanding of the differences among the various gyroscope-based heading methods, in Figure 4, we show the angular turning rate as computed by each one of them. It is easy to see how the vertical component of the gyroscope ($\omega_{v}$) is still biased downwards, as we expect it to fluctuate around zero during straight path segments. This happens, despite our attempt to eliminate this sensor’s bias based on the initial stationary interval, and it indicates the time-varying nature of the true bias (and possibly additional sources of error, such as gyroscope scale factors or errors in the direction of gravity). Additionally, we see that the signed-magnitude turn rate ($\omega_{sm}$) is larger in amplitude and exhibits discontinuity, as expected. The profile of Modified $\omega_{v}$ demonstrates the detection of straight walking segments, which is leveraged for dynamic bias compensation in the angular turning rate.

### 4.4. Sequential Processing of Tri-Axis Sensors

**Figure 5:**
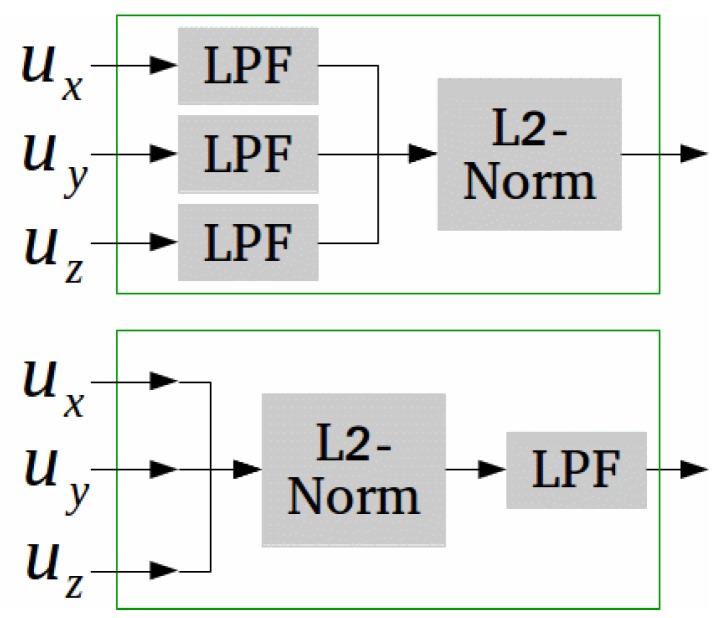
Given a general three-component signal, $u\left(t\right)=\left(u_{x}\left(t\right),u_{y}\left(t\right),u_{z}\left(t\right)\right)$, there are two forms of concatenating magnitude calculation and low-pass filtering in the processing implementation.

Another analysis of interest, which is presented next, deals with a more general question of how to apply a sequence of processing steps on vector measurements—such as those obtained from tri-axis inertial sensors—when part of the processing is nonlinear. An important example for this is the computation of measurements magnitude when low-pass filtering (LPF) is required: there are two forms in which this can be implemented, as illustrated in Figure 5. Theoretically, these two processing sequences are not equivalent, since the magnitude operator ($L_{2}$-norm of a vector) is nonlinear.

**Figure 6:**
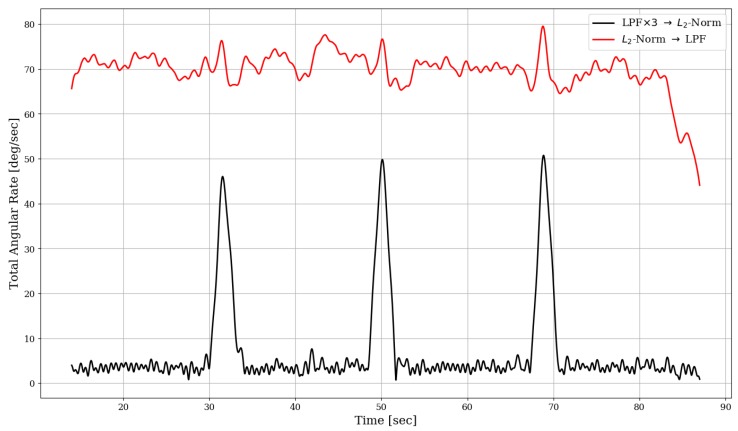
Magnitude of angular rate vector, comparing two different forms for sequential processing of the gyroscope raw data.

Figure 6 demonstrates this for the case of gyroscope processing, based on one of the experiments we conducted, which included three significant turning events. Comparing the resulting rate magnitude signals, it is clear that the order of computation in this case affects the result dramatically. In particular, the option of using tri-axis low-pass filtering prior to magnitude calculation (the black line in Figure 6)—compared to a single-axis filtering of the raw signal’s magnitude (the red line)—seems to produce dominant peak at each turning events; these peaks are very distinctive with respect to the “base” value of the signal.

Additional examples for which the processing sequence is important, in the context of pedestrian navigation, are:

- the calculation of gravity direction based on the accelerometer, as was described in Section 2.2, which involves the normalizing operation along with low-pass filtering;
- applying the magnitude calculation and LPF processing for the accelerometer (e.g., for step detection) or for the magnetometer (e.g, for magnetic fingerprint-based indoor navigation);
- projecting the gyroscope measurements onto the vertical direction, as in Equation (33), which requires low-pass filtering as well.

## 5. Conclusions

The framework derived in this paper considers the direction of gravity as a central element in PDR algorithms, particularly for pedestrian heading determination. A detailed derivation of two approaches for estimating the gravity direction was presented, along with four methods to compute relative heading angle. While the accelerometer-based gravity direction gives an approximate but robust solution, the fusion-based one is designed to achieve higher accuracy by tracking the rotational motion within the walking cycles, but requires a more complex tuning of its parameters. The experimental results shown here—as well as those from our initial research, [4]—indicate that the fusion-based gravity direction may indeed improve the heading accuracy, compared to the simpler accelerometer-based method, but its robustness is subject to a wider range of experimental testing. Potential improvement of the filter may be achieved by considering a more realistic setting of the measurements uncertainty, such as data-dependent model for the variance parameter.

Regarding the various methods for heading computation that were derived and compared in this work, we conclude the following:

1. When implementing gyroscope-based approaches, one must incorporate additional information or assumptions in order to mitigate its natural drifting. The method we proposed in this context makes use of straight path segments in order to dynamically adapt the estimated bias of the horizontal turning rate. This relatively simple model appears to produce reasonable heading accuracy throughout the entire experiment.
2. Using magnetic sensor for heading computation can be implemented by directly employing the gravity unit vector, as suggested in Section 3.5. This method showed superior performance compared to the gyroscope-based methods in the tested outdoor scenario, though it might be subject to more significant magnetic disturbances when applied indoors.
3. Considering the vertical projection of the gyroscope versus the magnitude of the angular rate vector, it seems that the former is more accurate as a means for computing the heading angle. Moreover, it naturally allows for distinguishing between left and right turns.

Finally, we have also demonstrated how the order of sequential processing—particularly magnitude calculation and low-pass filtering of gyroscope data—can be crucial for achieving the desired output signal. We conclude that such subtle design choices must be treated carefully when dealing with PDR algorithms in general.

## Figures and Tables

**Table 1:** Summary of the various methods proposed in the paper

	Method Title	Section
**Gravity Direction Estimation**	Accelerometer-based	Section 2.2
	Fusion-based	Section 2.3
**Heading Computation**	$\omega_{v}$-based	Section 3.2
	$\omega_{sm}$-based	Section 3.3
	Modified $\omega_{v}$-based	Section 3.4
	Magnetic-based	Section 3.5

**Table 2:** Comparison results summary for all combinations of the various methods, showing the error in heading angle during the last segment of the 85-second scenario (N.C. values indicate errors that are quickly drifting and non-comparable).

		Gravity Direction Method	
		Accelerometer-Based	Fusion-Based
**Heading Method**	$\omega_{v}$-Based	N.C.	N.C.
	$\omega_{sm}$-Based	N.C.	N.C.
	Modified $\omega_{v}$-Based	<15 [deg]	9 [deg]
	Magnetic-Based	≈6 [deg]	fluctuating

## Abbreviations

The following abbreviations are used in this manuscript:

PDR	Pedestrian Dead Reckoning
NED	North–East–Down
LPF	Low-Pass Filter / Filtering
GNSS	Global Navigation Satellite System
WMM	World Magnetic Model
